# Analyzing the Behavior and Growth of Cycling in Four North American Cities Before, During, and After the COVID-19 Pandemic

**DOI:** 10.1177/03611981231157396

**Published:** 2023-03-17

**Authors:** Eduardo Adame Valenzuela, Pierre Barban, David Beitel, Luis Fernando Miranda Moreno, Van-Thanh-Van Nguyen

**Affiliations:** 1 Department of Civil Engineering and Applied Mechanics, McGill University, Montréal, Québec, Canada; 2 Eco-Counter Canada/USA, Montréal, Québec, Canada

**Keywords:** data and data science, data analysis, trip purpose data, bicycles, traveler behavior

## Abstract

The paper highlights the changes in cycling patterns and ridership trends across 12 years (including the COVID-19 pandemic) in Montreal, Vancouver, Ottawa, and New York. Using data from 17 bicycle counting stations, changes in the dynamics of daily and weekly profiles before and during pandemic were determined. Additionally, the ridership demand evolution across the years was explored using models that controlled for variations in the weather. All the studied bicycle facilities experienced changes in the daily and hourly patterns in 2020 (the first year of the pandemic), tending toward recreational purposes. Significant growth in bicycle activity during the first year of the pandemic has been found, but trends for the following years (2021 and 2022) have not been studied. This study found that all counting sites located on cycling facilities primarily used for utilitarian purposes experienced a growth in ridership during 2020. Ridership on utilitarian corridors in Montreal and New York City grew considerably during the pandemic before stabilizing in 2021 and 2022. The same counting sites rapidly reverted to utilitarian hourly and daily patterns in 2021. The mixed-utilitarian bicycle facilities in Ottawa and Montreal shifted toward more recreational uses during the pandemic, though ridership did not grow in 2021 and 2022. All the counting sites in Vancouver shifted toward mixed use during the first year of the pandemic and did not show any clear signs of reverting to their utilitarian patterns.

During the last two decades, several North American cities have seen an increase in bicycle ridership, which in part can be related to investment in the cycling infrastructure (*1*). With energy and climate change concerns, cycling is seen as one of the most sustainable transportation modes. Cycling not only reduces fossil fuel consumption and related emissions but also helps to improve public health and wellbeing (*2*). Bicycle trips represent a relatively small modal share compared with the automobile in North American cities; however, a significant increase has been observed in several cities in recent years. The COVID-19 pandemic in 2020, had a major impact on transportation demand. While public transportation mode share reduced significantly, bicycle mode share growth became more pronounced (*3*).

During the pandemic, bicycle infrastructure played a critical role in cities, offering an alternative transportation mode for commuters with public health and safety concerns related to public transit systems. A positive correlation between bicycling sharing system (BSS) trips and the number of new daily COVID cases in New York City, as shown by Teixeira and Lopes, suggests a possible modal shift from the subway system toward the CityBike system (*4*). Bicycle infrastructure also provided the general population with the opportunity to safely participate in recreational activities during the pandemic (*5*). Bicycle infrastructure helped ensure access to destinations and activities during the pandemic with minimum economic and environmental impacts. Accordingly, cycling has emerged not only as a sustainable but also a resilient mode of transportation (*6*, *7*).

Given the central role of cycling as a sustainable mode of transportation, a large body of related literature has been published in recent years, documenting key issues that limit cycling participation, such as road safety, the lack of infrastructure, or the impact of weather (*8*, *9*). A few papers have also looked at the cycling ridership evolution before and during the pandemic (*10*, *11*). Most research explored the impact of the weather before the pandemic or the challenges that the pandemic posed to bike-sharing systems, with significant changes observed in the usage patterns of shared-mobility bicycle services (*4*, *6*, *12–16*). These works are important in that they study the resilience of bike-sharing systems. However, they do not reflect a complete picture of bicycle infrastructure usage and demand during these critical pandemic years. Buehler and Pucher investigated the impact of the pandemic on cycling in various cities and countries of Europe, North America, and Australia (*3*). Using data from 2019 and 2020, they attempted to establish overall trends as well as variations over time.

Despite the significant amount of work in recent years, to the best of the authors’ knowledge, very little work has explored long-term trends and detailed patterns of bicycle usage and demand before and during the pandemic in cities in North America. A significant growth in bicycle activity was observed in the first year of the pandemic, but there is less knowledge of trends in 2021 and 2022 (*3*). Furthermore, past research on cycling trends has not controlled for the potential impact of the weather on seasonal/yearly variations, leading to results that could either under- or overestimate cycling growth.

This paper illustrates the long-term bicycle patterns and trends before and during the pandemic across four major cities in North America (Montreal, Vancouver, and Ottawa in Canada, and New York City in the United States) during 2010/2022. The specific objectives were: (i) to propose a methodology to characterize the temporal dynamics of daily and weekly ridership profiles to identify trends in utilitarian patterns, and (ii) to investigate the ridership trends across seasons and years in four cities while controlling for weather variations. Understanding the behavioral and growth trends occurring in each city may provide city planners and authorities with greater knowledge of current and future cyclist demand. This knowledge could be used to inform modifications to existing facilities and the creation of new active transportation routes. Additionally, controlling for weather allows for fair comparison of cities located in different climates.

The following sections review the existing literature, give a summary of the methodology, a description of the case cities and the corresponding data, and finally an analysis of the results to better understand the temporal variations in cyclist behavior and demand across the cities during the pandemic.

## Literature Review

In investigating the changes in travel behavior by analyzing the census data in Seoul’s metropolitan area, Choi et al. discovered that bike trips increased in both duration and frequency from 2002 to 2006 (*17*). Pucher et al. stated that cycling levels stagnated in the United States from 2001 to 2009 based on the results of the National Household Travel Surveys (*18*). When analyzing the trends in demand of London’s bike-sharing system, Chibwe et al. found that the number of trips increased throughout the years as the unemployment rate decreased, which could mean that most of the scheme’s trips were utilitarian in nature (*11*). Ledsham et al. analyzed the results of surveys by using generalized structural equation models to better understand the factors influencing the amount of utilitarian and recreational trips in the suburbs of Toronto (*19*). Stinson et al. created statistical models capable of estimating the amount of utilitarian and recreational trips in the city of Los Angeles; nonetheless, no research on temporal changes in bike activity usage patterns throughout the years was found (*20*).

In 1995, Nankervis compared the daily counts of cyclists attending an Australian university with a weather condition index, which comprised data on the force of the wind, the maximum daily temperature, and the presence of rain (*21*). Although he did find a relationship between the variables, the magnitude of the influence was weaker than first assumed. However, the author mentions that the study focused on student behavior, which may not be representative of the general population. This change in commuting behavior may be explained by changes in the perception of safety that cyclists experience in adverse weather conditions (*22*). Interestingly, such behavior could be affected not only by current meteorological conditions, but also by the weather forecast (*23*). By applying a loglinear regression model to automatic bike counts, Thomas et al. found that temperature had the greatest influence, although wind speed and hours of sunshine and precipitation did have a significant effect; they also found that favorable weather could hide a downward trend in bicycle usage (*24*). Miranda-Moreno and Nosal found that temperature had a notable positive effect and humidity a negative effect on bike counts, whereas precipitation could have a significant lagged negative effect of up to 3 h on the observed counts (*25*). They also discovered that above a certain threshold, cyclists’ desire to bike starts to decline in line with higher temperatures. The thresholds were calculated to be at 28°C, 28°C, 30°C, and 27°C in Montreal, NYC, Seattle, and Austin, respectively (*25*–*28*). This indicates that the magnitude of the weather effects on cyclist demand will vary from city to city, as also noted by Goldmann and Wessel (*29*). The magnitude of the effect also varies depending on the use of the bike route. Zhao et al. found that weekday users of a bike train in Seattle were more resilient to weather conditions than their weekend counterparts (*27*). Also in Seattle, Niemeier discovered a greater variability of counts in the afternoon peak (from 3:30 to 6:00 p.m.) and stated that this could be partially attributed to the presence of nonutility cycling trips (*30*). Miranda-Moreno and Nosal in their study of the data from automatic counters in Montreal found that recreational locations were more sensitive to weather (*25*). Wessel also found that the effects of lighting conditions differ between utilitarian, recreational, and mixed-type users (*31*). Pazdan et al. classified nine bicycle corridors in the city of Kracow (*32*). They then added each site’s classification as an independent categorical variable in a regression model of bike counts; this was not found to be significant. When determining the influence of weather variables, studies have used regression models such as linear and loglinear (*24*, *29*), square root (*32*), loglinear in absolute and relative models (*25*), and negative binomial with log identity (*23*, *33*). Although those models dealt with the nonlinear relationship of temperature by using squared terms, and of precipitation by categorizing that variable, other studies have opted to use general additive models (*26*, *28*, *33*) that, instead of assigning a constant coefficient, determined the influence of each variable by function. When dealing with time-series data, it is important to control for autoregressive temporal effects (*27*). Some studies have addressed this issue by using auto-lagged effects (*26*, *34*) and by transforming the data using a nine-term average of the counts (*27*, *35*). Others have studied the use of autoregressive integrated moving average (ARIMA) models to account for this temporal correlation between observations (*32*, *36*).

In investigating the effects of the pandemic on cycling demand, Buehler and Pucher investigated the overall trends in cities across Europe, the Americas, and Australia by using automatic counter data (*3*). The authors concluded that cycling increased from 2019 to 2020 in most of the studied cities. Nikiforiadis et al. investigated the impacts of the pandemic on the perception of bike-sharing systems in the city of Thessaloniki in Greece (*16*). A field survey found that the pandemic made the city’s BSS a more attractive option, which could become the preferred commuting mode for current car passengers and users already registered in the system. In London, Heydari et al. compared the observed cycle hires in the city’s BSS with estimated counts had the pandemic not occurred (*15*). They found that although counts initially dropped in the initial months, they rapidly bounced back to expected levels, which is a strong indication of the system’s resiliency. The authors also noted an increase in the duration of trips, which could have been caused by a shift from public transit users to the BSS. Similarly, Shang et al. discovered an increase in the average duration of bike-share trips in Beijing during the pandemic (*37*). Wang and Noland analyzed data from New York City’s subway and bike-sharing systems, comparing the daily counts from 2019 to 2020 while controlling for the effect of weather (*7*). They concluded that both systems saw an initial decrease in riders during the first months of the pandemic. However, by September 2020, the BSS had nearly recovered to prepandemic levels, whereas the subway’s rider counts remained low. Through a face-to-face survey, Nguyen and Pojani reported an increase in recreational cycling in the city of Hanoi, Vietnam (*38*). They also stated that income and age were not significant factors in the decision to make more recreational trips and that most people adopted cycling as a way of increasing or maintaining their level of physical activity and socializing in an infection-safe environment.

## Methodology

**Figure 1.**
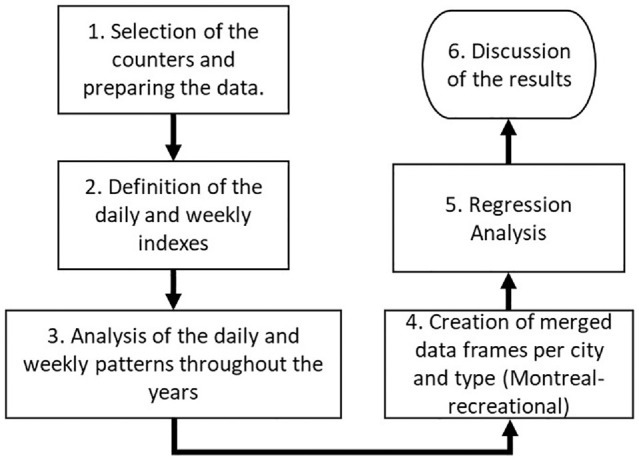
Methodology flow chart.

The methodology consisted of six steps as shown in Figure 1.

The first step was selection of the bicycle counting sites to be used in the research. The selection process was based on the following criteria: (i) the counters should be located on main corridors with few or no alternative routes; (ii) the counters should have historic, long-term, relatively complete count data of at least 8 years; (iii) the facilities should exhibit a mainly utilitarian pattern in the years preceding the pandemic; and (iv) the counters should be located on corridors with high annual average daily counts.

As a second step, each counting site was classified per day as utilitarian, recreational, or mixed based on their daily and weekly patterns by using the indexes defined in Equations 1 and 2, which were developed in a similar way to those presented by Miranda-Moreno et al. (*39*). The hourly index is defined as follows:

(1) $$I_{\mathrm{AM}/\mathrm{Noon}}=\frac{V_{\mathrm{peak}}}{V_{\mathrm{out}}}=\;\frac{\frac{1}{2}\sum_{i=7}^{8}V_{i}}{\frac{1}{2}\sum_{i=11}^{12}V_{i}}$$

where $I_{\mathrm{AM}/\mathrm{Noon}}$ is the hourly index computed as the ratio of $V_{\mathrm{peak}}$, or the average peak-hour volume, from 7:00 to 9:00 a.m.; and $V_{\mathrm{out}}$ is the average off-peak volume from 11:00 a.m. to 1:00 p.m. Here, *V_i_* stands for the number of counts at hour *i*, and *n* is the number of hours in the period (2 h for the AM peak from 7:00 to 9:00 a.m. and 2 h for the noon peak extending from 11:00 a.m. to 1:00 p.m.).

The weekday versus weekend is defined as

(2) $$I_{\mathrm{wd}/\mathrm{we}}=\frac{D_{\mathrm{wday}}}{D_{\mathrm{weekend}}}=\frac{\frac{1}{5}\sum_{i=1}^{5}D_{i}}{\frac{1}{2}\sum_{i=6}^{7}D_{i}}$$

where $I_{\mathrm{wd}/\mathrm{we}}$ is the daily index computed as the ratio of $D_{\mathrm{wday}}$, or the average daily volume from Monday to Friday (*i* = 1 on Monday, *i* = 2 on Tuesday, etc.); and $D_{\mathrm{weekend}}$ is the average daily volume from Saturday to Sunday. These formulas gave a unique AM/noon index for each day of the historic data, excluding weekends, and the same weekday/weekend index for every day during the same week.

In the third step, the days were classified using a similar process to that presented by Wessel (*23*). If both indexes were greater than 1, the day was considered to have a utilitarian pattern; if both indexes were less than 1, it was considered recreational, and to be mixed for every other case, that is,

If $I_{\mathrm{AM}/\mathrm{Noon}}>1$ and $I_{\mathrm{wd}/\mathrm{we}}>1$ then day type = utilitarian;
If $I_{\mathrm{AM}/\mathrm{Noon}}<1$ and $I_{\mathrm{wd}/\mathrm{we}}<1$ then day type = recreational;
Otherwise,
Day type = mixed.

Then, for every year, the percentage of utilitarian, recreational, and mixed days were calculated (Figures 3 to 7).

In the fourth step, all counting sites were classified into four possible groups—the same classifications used in the research by Miranda-Moreno et al. (*39*). If the counting site had 60% or more of its days classified as utilitarian, then the site was considered to be used for purely utilitarian purposes. If 60% or more of the days were recreational, then the site was classified as a purely recreational location. The remainder were classified as mixed and divided into two groups: mixed-utilitarian, when the number of utilitarian days was greater than the number of recreational days, or mixed-recreational for the inverse case, that is,

If utilitarian days ≥60% of all days in the year, then site type = utilitarian;
If recreational days ≥60% of all days in the year, then site type = recreational;

Otherwise,

If utilitarian days > recreational days, then site type = mixed-utilitarian; and
If utilitarian days < recreational days, then site type = mixed-recreational.

Afterwards, for the all counting data from the same city and of the same type (e.g., Montreal-utilitarian, Montreal-mixed) their total daily counts were paired with the daily measured weather factors, the daily stringency index, a holiday dummy variable (1 if the day was a national holiday, 0 if not), a bridge dummy variable (1 if the day was a Friday after a holiday or a Monday before a holiday, 0 if not), a location variable (site ID), and the year the count was taken. Similar weather variables, such as the maximum- and average daily temperatures, were compared and those with the highest correlation to the counts, or with the greatest explanatory power, were kept for future analysis. A further correlation analysis was subsequently carried out to ensure independence of the explanatory variables. If there was a high correlation between two of them (i.e., ≥0.5), the variable with the greater correlation to the counts was kept.

In the fifth step, a regression analysis was carried out by fitting each data frame, optimized to eliminate any correlated or nonsignificant variables, to two different regression models that have been widely used in similar studies: loglinear and the negative binomial with log identity.

In the final step, the yearly coefficients of the models were analyzed to determine whether the cities were experiencing an overall growth in cycling demand independent of weather conditions.

## Data

### Counters

The study used bike counts, from counters manufactured by Eco-Counter, which were installed in cities with an open data access policy. The study only used counts that were classified as “bike” by the counter algorithm. A first selection was made by identifying the counting locations on cycling routes with few (or no) alternatives. Therefore, counting locations on bridges, underpasses, and major cycling routes were prioritized. This reduced the likelihood that temporal bike lanes, also called “pop-up” lanes, or new infrastructure in general, would affect growth trends at the selected counting location. In the first stage, only the counters that showed a small number of outliers in their historic data were selected. Additionally, all locations with a low mean daily count compared with the other sites of the same city were dropped. This generated a total of 17 study sites. The extreme values from these sites were identified using a visual time-series analysis and manually replaced by a null value. For the cases in which three consecutive days or fewer had null values, the counts were linearly interpolated. All other null values were deleted from the analysis. There were some additional deletions of data for specific counters, which will be explained in the following sections.

The data spanned from the installation of the counter until June 30, 2022. Table 1 gives the summary statistics for all the daily cyclist counts collected at each of the studied counters. Although the number of total sites per city might be smaller than the ideal, this allowed for a more relevant dataset and for careful inspection of the counts. However, the low number of sites included was a limitation of this study.

**Table 1.** Summary Statistics of the Daily Cyclist Counts for Each Counter (Units in Cyclist Per Day)

Site	Mean	Median	Maximum	Minimum	Standard deviation
New York City					
NY1	4,830.46	4,819.5	10,940	162	2,461.34
NY2	3,702.06	3,689	8,295	21	1,793.27
NY3	3,953	3,907	9,466	117	2,082.66
Montreal					
MT1	1,137.9	835	5,857	0	1,122.83
MT2	2,725.09	2,324.5	9,772	0	2,200.08
MT3	2,695.92	2,174	11,092	0	2,358.26
MT4	1,133.45	930	5,337	0	1,014.53
MT5	1,451.16	1,147	5,305	0	1,315.37
MT6	2,347.46	1,805	8,812	0	2,100.59
Ottawa					
OT1	830.57	619	3,340	0	774.35
OT2	994.04	650	4,128	2	948.42
OT3	426.88	272	2,350	0	470.62
OT4	528.08	390	2,724	0	495.21
OT5	930.96	751	3,284	0	865.62
Vancouver					
VN1	3,621.61	3,151	10,187	0	2,272.09
VN2	1,972.62	1,440	8,823	0	1,716.27
VN3	3,584.98	3,244.5	10,129	56	2,084.33

#### New York City

New York was the most populous city in this study, with a population of 8,550,405 in 2015 and a density of 10,474.7 residents/km^2^ (*40*). The city experiences warm, humid summers and mild to cold winters (*41*). By 2018, the city had installed 1,240 mi of bike lanes and had a daily average of 490,000 cycling trips. In 2022 the borough of Manhattan was awarded a rating of 54 out of a possible score of 100 points according to the PeopleForBikes’ City Rating (*42*), ranking the area 11th among large cities. The selected counters were the Williamsburg Bridge path (NY1), the Ed Koch Queensboro Bridge Shared Path (NY2), and the Manhattan Bridge Display Bike Counter (NY3). The bike counts from the Manhattan Bridge pedestrian path were added to the NY3 site to account for any cyclists that may have changed routes because of partial or total closure of the cycling path.

#### Montreal

In 2021 Montreal had a population of 4,291,732 and a density of 919 residents/km^2^ (*43*). Currently, there are 889 km of bike lanes in the city (*44*). In the City Ratings, Montreal was awarded a rating of 65 out of a possible score of 100 points, ranking it 1st among the large cities of North America (*42*). The selected counters were located on the Jacques-Cartier Bridge (MT1), Rue Rachel near the Papineau intersection (MT2), Boulevard Maisonneuve near the intersection with Rue Peel (MT3), Côte Sainte-Catherine near the intersection with Rue Stuart (MT4), Avenue du Parc near the intersection with Rue Duluth (MT5), and Rue Berri near the intersection with Rue Ontario (MT6). The 2020 counts of the MT6 site were deleted owing to a construction project that reduced the access to Rue Berri.

#### Ottawa

The Ottawa metropolitan area had an estimated population of 1,135,014 and a density of 243.3 residents/km^2^ in 2021 (*43*), which made it the smallest and least dense city in this study. The city was awarded a rating of 51 out of a possible score of 100 points in the PeopleForBikes City Ranking 2022, ranking it the 13th best place to bike among the large cities of North America (*42*). The selected counters were the NCC Eastern Canal Pathway Colonel By (OT1), one on Avenue Laurier near the intersection with Metcalfe (OT2), the Trillium Bayview (OT3), the Trillium Gladstone (OT4), and the NCC Alexandra Bridge Cycle Track counter (OT5).

#### Vancouver

The metropolitan area of Vancouver had a population of 2,642,825 and a density of 918 residents/km^2^ in 2021 (*43*). With a cycling network of 325 km in 2018 (*45*), the city was awarded a raiting of 56 out of possible score of 100 points in the 2022 City Rankings (*42*), which ranked it the 6th of all large cities in North America. The selected counters were located on the Seawall path near Science World (VN1), on the Seawall path near the Creekside Community Centre (VN2), and on Burrard Street near the intersection with Cornwall Avenue (VN3). Because of the presence of anormal counts during 2012, that year of data were deleted from the VN1 site.

### General Analysis

**Figure 2.**
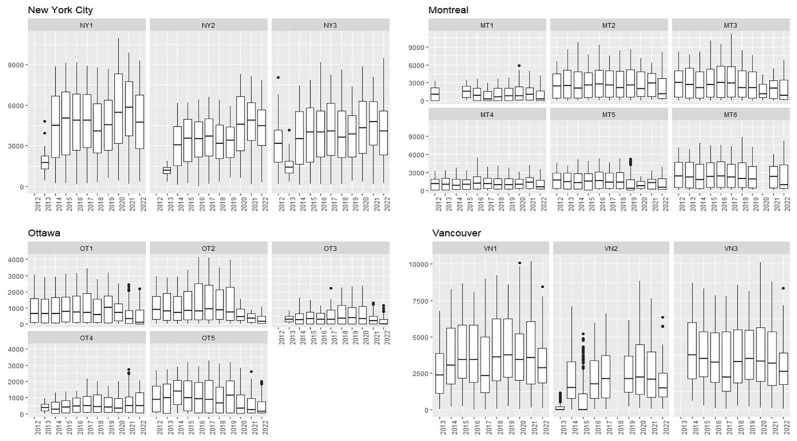
Boxplot of the daily cyclist counts from each of the studied sites.

Analyzing the boxplots presented in Figure 2, the New York sites’ median values for daily cyclist counts do seem to increase slightly in the last years. Looking at the Montreal counters, there’s no visible trend citywide, which is the similar case in Vancouver. Most of the counters in Ottawa seem to show a downward trend in the last years, even before the start of the lockdown measures. However, it would be wrong to assume, based on these plots, that cycling demand in the cities have decreased in the last years. As noted in the literature, it is important to control for any external variables, such as the general weather and the lockdown measures that could hide the inherent trend of cycling demand before making any assessment about future investments.

### Weather Variables

The weather data were obtained through World Weather Online’s application programming interface. For each city, the measured weather data were recovered from the station closest to the centroid of all the counters in that city. The initial weather variables extracted for analysis were daily maximum and average temperature (°C), daily average and maximum wind speed (km/h), daily total precipitation (mm), daily average and maximum precipitation intensity (mm/h), daily average and maximum humidity (%), as well as the daily percentage of the sky that was obscured by clouds, also known as cloud cover.

### COVID Variable

Ritchie et al. calculated a per-day stringency index, which is the mean score of nine different metrics: school closures, workplace closures, cancellation of public events, restrictions on public gatherings, closures of public transport, stay-at-home requirements, public information campaigns, restrictions on internal movements, and international travel controls (*46*). The possible values have a range from 0 to 100 (where 0 means there are no restrictive measures in place and 100 equates to the strictest response by the authorities).

## Results

### Descriptive Analysis: Behavioral Patterns by Indexes

**Figure 3.**
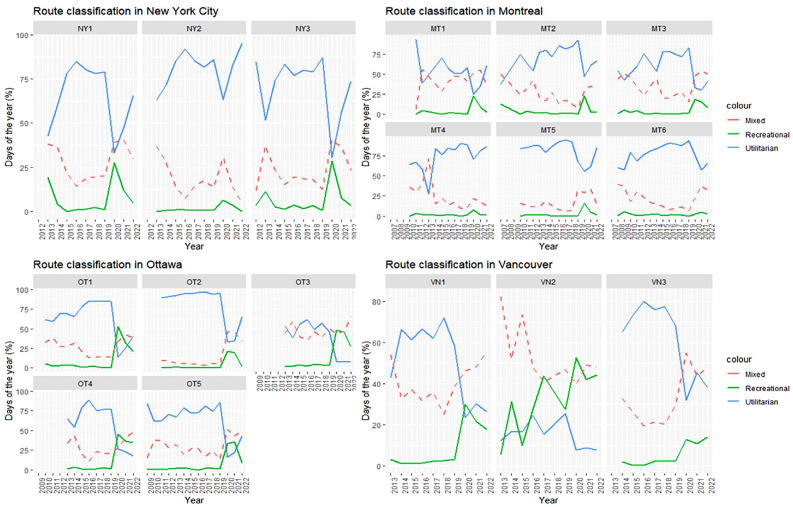
Temporal changes in usage of the facilities.

From Figure 3 it can be observed that most of the selected bicycle corridors exhibited a utilitarian pattern before the start of the pandemic. During the pandemic (2019 to 2022), the study facilities experienced a shift toward more mixed or even fully recreational patterns. This was expected as the lockdown measures required people to work from home, which diminished the number of commuter trips. The biggest impact was seen in 2020 when 6 of the 17 sites shifted from utilitarian to mixed, and three others shifted from utilitarian to recreational use. However, 2021 and 2022 showed some recovery toward mixed-utilitarian or purely utilitarian patterns in most cases, which could suggest an eventual return to the previous uses in most of the study corridors.

Two of the New York counting sites had some of the biggest declines in utilitarian days during 2020. Nonetheless, all three of them experienced a rapid recovery toward the patterns observed before the start of the lockdown measures. Ottawa also experienced sharp drops in utilitarian days during the pandemic. However, unlike New York City, the recovery toward previous patterns occurred more slowly in three of the sites. However, in the other two Ottawa sites, both located on the Trillium path, the change toward mixed patterns seemed to be permanent, as the number of utilitarian days stagnated after 2020. Ottawa also registered the highest percentage of recreational days compared with the other study cities.

**Figure 4.**
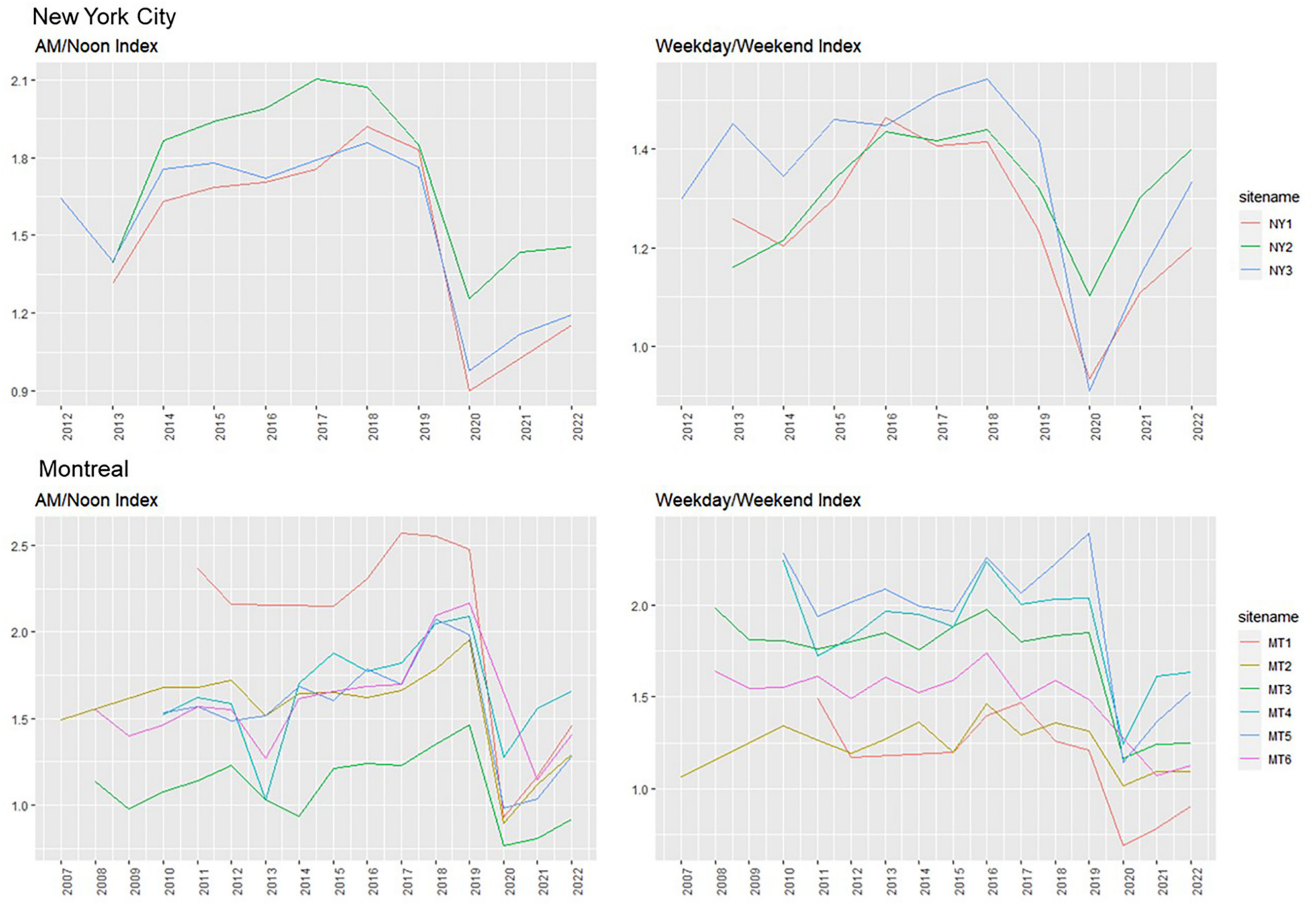
Yearly variation of the indexes in New York and Montreal.

Montreal was the city with the most consistent patterns throughout the pandemic. Although all the locations showed a dip in 2020, most still mainly experienced utilitarian days. Only two saw a shift in 2020 toward mixed patterns. These patterns were more closely related to those of New York City, although with less pronounced 2020 dips (Figure 4).

**Figure 5.**
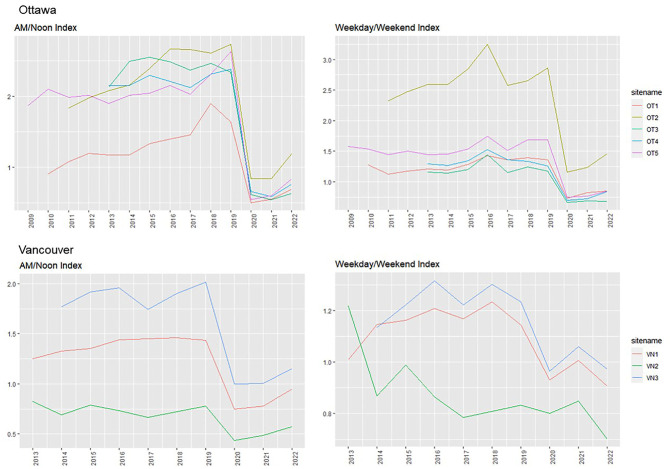
Yearly variation of the indexes in Ottawa and Vancouver.

Vancouver showed a different behavior, being the only city with more mixed-pattern days across all its counting sites. It is noteworthy that the VN2 location trended toward recreational use before the start of the pandemic. The other two sites were like those seen in Ottawa, where utilitarian patterns shifted toward mixed and did not show any signs of shifting back. In both, there was a significant decline in utilitarian days in 2020, then a small increase in 2021, followed by a smaller dip in 2022. The indexes in this case were interesting (Figure 5): Vancouver was the only city where the weekday/weekend ratio continued to decline in 2022, even though the AM/noon peak ratio was increasing slightly. This mismatch in tendencies explained the mixed classification.

### Regression Analysis: Yearly Trends After Controlling for Weather

A regression analysis was carried out for every type of route in the four cities. Each data frame was optimized by eliminating all correlated variables and by only keeping those variables that were significant at a 95% confidence level. Owing to space limitations, only the coefficients and results for the negative binomial models of the utilitarian facilities are shown in Table 2, since their *R*^2^ score showed a lower variability. The coefficients calculated by the models, which were built using R functions, were depicted as the percentage change in counts through time compared with a base year, which was the first year of available data for every individual dataset.

**Table 2.** Coefficients of the Negative Binomial Models

City	New York			Montreal			Ottawa			Vancouver		
Coefficients	Estimate	SE	Sig.	Estimate	SE	Sig.	Estimate	SE	Sig.	Estimate	SE	Sig.
(Intercept)	7.68	0.03	***	6.43	0.05	***	5.53	0.06	***	8.25	0.07	***
Site_cat2	−0.25	0.01	***	−0.05	0.01	***	0.44	0.02	***	–	–	–
Site_cat3	−0.20	0.01	***	−1.02	0.01	***	−0.35	0.02	***	–	–	–
Site_cat5	–	–	–	−0.73	0.01	***	0.00	0.02		–	–	–
Site_cat6	–	–	–	−0.20	0.01	***	–	–	–	–	–	–
Maximum daily temperature	0.09	0.00	***	0.08	0.00	***	0.08	0.00	***	0.11	0.00	***
Squared maximum daily temperature	0.00	0.00	***	0.00	0.00	***	0.00	0.00	***	0.00	0.00	***
Maximum wind speed	−0.01	0.00	***	−0.01	0.00	***	−0.01	0.00	***	–	–	–
Average wind speed	–	–	–	–	–	–	–	–	–	−0.03	0.00	***
Precip_cat2	–	–	–	−0.09	0.01	***	−0.07	0.02	***	–	–	–
Precip_cat3	–	–	–	−0.51	0.03	***	−0.50	0.04	***	–	–	–
Precip_cat4	–	–	–	−0.45	0.12	***	−0.36	0.09	***	–	–	–
Precip_cat5	–	–	–	−0.63	0.30	*	−0.83	0.21	***	–	–	–
Average daily precipitation	−0.08	0.00	***	–	–	–	–	–	–	–	–	–
Average humidity	–	–	–	–	–	–	–	–	–	−0.01	0.00	***
Average cloud cover	0.00	0.00	***	−0.01	0.00	***	−0.01	0.00	***	−0.03	0.00	***
Total depth of snow (cm)	−0.03	0.00	***	−0.05	0.00	***	−0.02	0.00	***	−	−	−
Stringency index	0.00	0.00	**	−0.01	0.00	***	−0.01	0.00	***	−0.18	0.05	***
Holiday dummy 1	−0.45	0.02	***	−0.75	0.05	***	−0.40	0.04	***	−0.21	0.09	*
Bridge dummy 1	0.12	0.03	***	0.23	0.06	***	–	–	–	0.06	0.03	*
Day of the week 2 (Tuesday)	0.04	0.01	***	0.04	0.02	**	–	–	–	0.05	0.03	.
Day of the week 3 (Wednesday)	0.07	0.01	***	0.04	0.02	**	–	–	–	0.01	0.03	n.s.
Day of the week 4 (Thursday)	0.03	0.01	**	0.04	0.02	*	–	–	–	−0.06	0.03	*
Day of the week 5 (Friday)	−0.01	0.01	n.s.	−0.05	0.02	**	–	–	–	−0.18	0.03	***
Day of the week 6 (Saturday)	−0.20	0.01	***	−0.56	0.02	***	–	–	–	−0.24	0.03	***
Day of the week 7 (Sunday)	−0.33	0.01	***	−0.64	0.02	***	–	–	–	–	–	–
Weekend_dummy 1	–	–	–	–	–	–	−0.49	0.01	***	–	–	–
Season_dummy (spring)	0.10	0.01	***	0.60	0.02	***	0.76	0.02	***	0.35	0.02	***
Season_dummy (summer)	0.15	0.01	***	0.71	0.02	***	0.80	0.03	***	0.37	0.03	***
Season_dummy (autumn)	0.15	0.01	***	0.89	0.02	***	0.82	0.02	***	0.07	0.02	**
Year 2009	–	–	–	0.16	0.05	***	–	–	–	–	–	–
Year 2010	–	–	–	0.41	0.04	***	−0.40	0.06	***	–	–	–
Year 2011	–	–	–	0.38	0.04	***	−0.09	0.06	n.s.	–	–	–
Year 2012	–	–	–	0.40	0.04	***	−0.01	0.06	n.s.	–	–	–
Year 2013	−0.06	0.04	.	0.47	0.04	***	−0.06	0.06	n.s.	–	–	–
Year 2014	0.05	0.03	.	0.46	0.04	***	−0.04	0.06	n.s.	–	–	–
Year 2015	0.14	0.03	***	0.54	0.04	***	0.10	0.06	.	−0.01	0.03	n.s.
Year 2016	0.19	0.03	***	0.63	0.04	***	0.03	0.06	n.s.	0.01	0.03	n.s.
Year 2017	0.20	0.03	***	0.66	0.04	***	0.18	0.06	**	−0.16	0.03	***
Year 2018	0.15	0.03	***	0.54	0.04	***	0.10	0.06	n.s.	−0.06	0.03	.
Year 2019	0.16	0.03	***	0.53	0.04	***	0.10	0.06	n.s.	−0.04	0.03	n.s.
Year 2020	0.33	0.03	***	0.97	0.05	***	0.60	0.09	***	0.04	0.03	n.s.
Year 2021	0.37	0.03	***	1.15	0.06	***	0.40	0.09	***	−0.02	0.03	n.s.
Year 2022	0.36	0.03	***	1.10	0.06	***	0.17	0.08	*	−0.12	0.04	**
2 × log-likelihood	−157,726.231			−40,893.404			−226,507.317			−49,342.23		
Akaike information criterion	157,790			40,945			226,573			49,394		

*Note*: Site_cat = variable representing the different counters in the analysis (e.g., Site_cat2 = MT2, Site_cat3 = MT3). Precip_cat = average daily precipitation grouped. Precip_cat base = no precipitation; Precip_cat2 = precipitation values in the 25% quantile; Precip_cat3 = precipitation values between the 25% and 50% quantiles; Precip_cat4 = precipitation values between the 50% and 75% quantiles; Precip_cat5 = precipitation values above the 75% quantile. Sig. = significance-level codes: “***” = *p* < .001; “**” = *p* < .01; “*” = *p* < .05; “.” = < .1; “n.s.” = Not significant; “–” = unused variable. SE = standard error.

The utilitarian bike facilities located in the cities of New York (NY1, NY2, and NY3) and Montreal (MT2, MT3, MT4, MT5, and MT6) showed consistent growth throughout the years studied, which increased in the first year of the pandemic. This could be attributed to the implementation of pop-up bike lanes, the closure of streets, or to the mode shift of public transport users toward biking resulting from transit service losses and greater concerns about infection in crowded vehicles. The trend seemed to stabilize in 2021 and 2022 for both cities, which could be a sign of the general recovery of the main public transport services in those cities. Ottawa’s utilitarian facilities (OT1, OT2, OT4, and OT5) also experienced consistent growth throughout the years. However, for the first half of the time frame, they showed lower levels of demand compared with the base year.

The mixed-utilitarian cycling corridors in Ottawa (OT3) and Montreal (MT1) told a different story, with a more erratic growth patterns than their utilitarian counterparts. Both showed a downward trend in demand, which was magnified during the first year of the pandemic. In the final years the decrease seemed to have stagnated in Ottawa and even reversed in Montreal. However, it should be noted that the data analyzed derived from only one counter in both cases, which means that this behavior represented a local situation and cannot therefore be generalized to the rest of the mixed-utilitarian sites across the cities. Future research should confirm whether this was a citywide- or simply a local trend.

All of Vancouver’s locations classified as different types. Site VN3 was the only utilitarian route in the analysis and showed stagnated growth, except in 2017 when it suffered a decrease in counts. As with the other utilitarian sites, growth increased in 2020. Nonetheless, VN3 was not capable of retaining the higher demand in the following years. Site VN1 was classified as mixed-utilitarian and showed a similar trend to that observed in the utilitarian facilities, with a constant growth throughout the years, except for 2017, and a stagnation in final last 2 years. As with the mixed-utilitarian corridors, these results may only represent the location of the counter and cannot be assumed to be the general situation of the city.

It is important to note that most of the models of the mixed-pattern corridors showed lower *R*^2^ scores than the utilitarian models because of the greater variability in the counts. These models were based on data from a single rather than a group of counters, which may explain the lower scores. Although beyond the scope of this paper, it is interesting to note that all utilitarian facilities across the four cities seemed to have stagnated or even suffered a decrease in counts during the years 2018 to 2019, just before the growth in counts caused by the pandemic.

**Figure 6.**
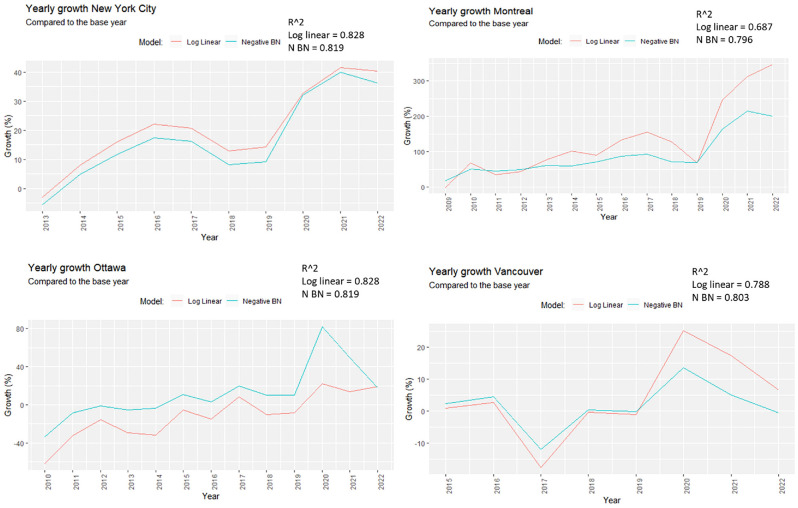
Growth in utilitarian bicycle facilities. *Note*: N BN = Negative Binomial.

**Figure 7.**
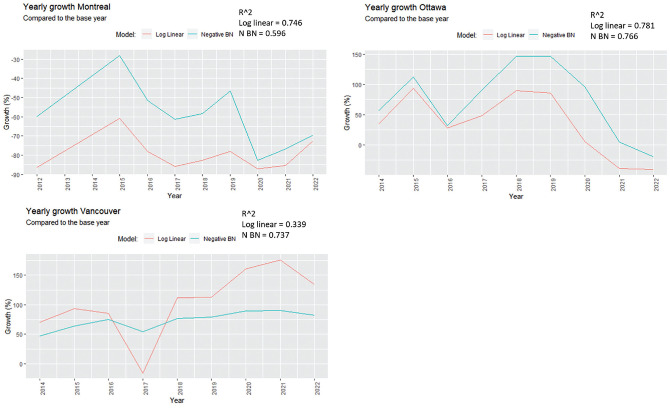
Growth in mixed-utilitarian bicycle facilities. *Note*: N BN = Negative Binomial.

Figure 6 illustrates the utilitarian facilities in the four cities and the mixed-utilitarian facilities are shown in Figure 7.

## Conclusions

This study investigated the long-term trends across cycling seasons before and after the pandemic using data from counting stations in four North American cities. It was observed that most of the utilitarian facilities in the four cities experienced changes in the daily and hourly patterns during the first year of the pandemic (2020), which saw greater mixed and recreational use of the facilities. This was related to the lockdown and work-from-home measures implemented in the study cities. However, no generalized pattern was evident.

Most of the utilitarian facilities experienced growth in the years before the start of the pandemic; the magnitude of the growth increased in 2020, even after controlling for weather variations across the years. This was perhaps related to the shift by public transport users to other modes including cycling during the pandemic. In 2022, the magnitude of the growth as well as the hourly and daily patterns seemed to revert to those of the prepandemic years. The utilitarian cycling corridors in Montreal and New York City grew considerably during the pandemic, before stabilizing in the 2021 and 2022; they also reverted more quickly to mostly utilitarian patterns after 2020. This could be explained by both places having the two biggest bike infrastructure networks among the studied cities and may therefore have a greater commuter cycling culture. Although the utilitarian facilities in these cities did suffer a sudden change during the pandemic, they do not seem to have been affected by it. They may have even benefited from it.

The mixed-utilitarian bicycle facilities shifted toward more recreational uses during the pandemic. One could attribute this to the increase of recreational riders during the pandemic. However, they do not seem to have experienced any growth in the last 2 years, which may indicate that these corridors lost commuters rather than gained recreational users. The changes in the patterns after the pandemic varied between the cities. Although the facilities in Montreal showed a tendency toward more utilitarian patterns and slight growth in 2021 and 2022, the growth in the mixed facilities of Ottawa and Vancouver stagnated during the same years and showed a tendency to remain mostly utilitarian or even shift to more recreational uses, respectively. In a general sense, these facilities lost commuters during and after the pandemic. Future research to confirm this finding by analyzing more counting sites of the same type is necessary.

Ottawa presented a slower recovery to prepandemic patterns in most of its sites. Vancouver presented a distinct situation compared with the other three cities: its utilitarian route did not show any major growth during the last few years, except for 2020. However, its mixed-utilitarian route grew constantly throughout the years. Furthermore, all the studied sites in this city shifted toward mixed use during the first year of the pandemic, none of which showed any clear signs of changing toward more utilitarian patterns. In general, the studied biking facilities in Vancouver seemed to have increased its recreational users. Further studies with data from more counters may be necessary to confirm this finding.

The next steps for this investigation will be to conduct the same methodology with the data from the second half of 2022 and incorporate a greater number of counting sites from other cities in North America. Furthermore, more advanced modeling settings (general additive and dynamic ARIMA regression models) will be tested to account for serial autocorrelation.
